# Estimation of Body Stature Using the Percutaneous Length of Ulna of an Individual

**DOI:** 10.7759/cureus.6599

**Published:** 2020-01-08

**Authors:** Humara Gul, Shahid Mansor Nizami, Muhammad Amjad Khan

**Affiliations:** 1 Anatomy, Abwa Medical College, Faisalabad, PAK; 2 Anatomy, Nishtar Medical University, Multan, PAK; 3 Anatomy, Bakhtawar Amin Medical and Dental College, Multan, PAK

**Keywords:** ulna, length, body height, correlation.

## Abstract

Objective

To explore the association between the percutaneous length of ulna and height of that individual and to formulate a gender-specific formula.

Methods

This is a cross-sectional study conducted at the Nishtar Medical University, Multan from May 1 2019 to July 1 2019. Length of ulna and body height were measured for 100 participants, including an equal number of males and females. Means of age, length of ulna and body height were compared between both genders. Regression coefficient, Pearson correlation coefficient and constant were calculated and regression formulae were formed for calculating height from length of the ulna for males, females, and the whole study group, separately. Data analysis was done using Statistical Package for the Social Sciences; version 23 (SPSS Inc., Chicago, IL); p≤0.05 was considered statistically significant.

Results

Pearson correlation coefficient, regression coefficient, constant were statistically significant for males, females, and the whole study group (p<0.001). Regression equations that were devised after analyzing the data to estimate the stature from ulna length are as follows: for the whole study group: 42.830+4.671 (length of ulna); for males: 70.369+3.698 (length of ulna); for females: 18.562+5.617 (length of ulna).

Conclusion

The length of ulna provides a reliable and relatively accurate means for estimating an individual’s height. Regression formulae devised will be of practical use for forensic scientists, anthropologists, archeologists, clinicians and anatomists for estimating the stature from the length of the ulna.

## Introduction

Measuring the height is vital for assessment of nutrition and growth, to predict pulmonary functions and to calculate the body surface area in children [1]. There is a consistent ratio among the growth of limbs and trunk and is comparative to the total height of the patient. However, these ratios are dependent on sex, age, and race of the individual. There are numerous reasons to establish an alternative method for estimation of the height, e.g. estimation of height is needed to be determined from bony fragments following mass casualties [2]. Precise measurement of body height along with body weight is needed to assess the nutritional status and pharmacokinetic parameters. A variety of physical disabilities make the accurate measurement of height in standing position nearly impossible [3]. Scientists have long faced the difficulty of associating length of long bones with body height. For this purpose, various methods have been used so far. Many formulae have been proposed to estimate height from the length of the long bone, but there have been problems in applying one population-specific formula on a different population [4]. For example, African people have relatively long bones of arms and legs, and the formula designed for that population is not appropriate for application on the Asian population [5].

One of the long bones of the forearm is ulna and it lies medially. There is a process called olecranon process on the proximal end of the ulna and this process forms the joint with the humerus. There is a styloid process at the distal end of the ulna. Olecranon process is easily palpable as it lies subcutaneously, and its position is determined by the extension of flexion at the elbow joint. With the elbow extended, the olecranon process is in line with epicondyles of humerus, but these three bony markings form an equilateral triangle when the elbow is flexed. Ulna can be palpated along its whole length. At about the eighth week of fetal life, the ossification of ulna starts. The fusion of the proximal epiphysis and the shaft occurs during the 16th year of life and the fusion of distal epiphysis and shaft occurs during the 20th year of life.

Length of ulna has been used to estimate the height of the individual and has been observed to be more consistent than long bones of the lower limbs [1-2]. Various researchers have proposed a linear regression of height with the percutaneous length of the ulna of the same person [6]. Estimating the height from hand length is of significant practical importance in anthropometry and inquiries of medico-legal cases [7]. Length of the ulna is more precise in formulating the regression equation to estimate height than the length of long bones of lower limbs such as tibia [8].

In spite of the importance of this correlation and its practical application, there is still a huge gap in this model for south Asian and Sri Lankan population. As the relationship between the growth of limbs and thorax is affected by race, age, and gender of the individual, it is nearly impossible to devise a universal formula to estimate the height. Therefore, there is the utmost need to devise a gender, age, and race-specific formula for estimation of stature. Therefore, the current study was intended to explore the association of percutaneous length of ulna and height of an individual, and to devise a gender-specific regression formula.

## Materials and methods

This cross-sectional study was performed at the Nishtar Medical University, Multan over a tie period of two months from May 1 2019 to June 30 2019. The study was approved by the ethical review board of the university. The study conducted by Ilayperuma et al. [5] was taken as a reference. We included 100 medical students in the study by nonprobability consecutive sampling technique. All the participants were second-year and third-year medical students with age ranging from 20 years to 27 years. The study population included an equal number of males and females; all the participants were healthy. Participants of the study belonged to both rural as well as urban areas and had different socioeconomic statuses. Students suffering from apparent physical deformity, history of growth hormone therapy in childhood, and those suffering from malnutrition were not included in this study.

All the participants were required to give written consent before the start of the study. Length of the ulna was measured as the displacement of the styloid process from the tip of the olecranon process. Length was measured with the elbow fully flexed. Length of the ulna was measured on the right and left side, and the mean was taken. Digital sliding caliper was used to measure ulna length. The height of every participant was measured with a standing-height measuring instrument. Height was measured from the vertex to the floor, when the participant was standing upright and the head was in the Frankfort plane. Height and length of all participants were measured at a specific time between 2:00 pm and 6:00 pm. All these measurements of all the participants were taken by the same person in order to minimize personnel errors.

Means of age, length of the ulna, and body height were calculated and compared between both genders by applying independent t-test. Pearson correlation and linear regression were applied to evaluate the association of ulna length with the height of the individual. Correlation coefficient, regression coefficient, and constant were calculated for the whole study group and for both genders, separately. In the end, regression equations were formed for calculating the estimated height from the length of ulna, for males, females, and the whole study group separately. Data analysis was done using Statistical Package for the Social Sciences; version 23 (SPSS Inc., Chicago, IL); p≤0.05 was considered statistically significant.

## Results

The mean age of male students was 23.26 ± 1.45 years and of female students was 23.48 ± 1.47 years and the mean age was comparable between both genders (p=0.454). The height of males ranged from 159 cm to 187 cm while the height of females ranged from 145 cm to 169 cm. The mean height of male students was 171.70 ± 8.05 cm and was significantly greater than the height of female students i.e. 157.30 ± 8.22 cm (p<0.001). Ulna length of male students ranged from 24 cm to 31 cm while the ulna length of females ranged from 23 cm to 27 cm. The mean ulna length of male students was 27.40 ± 2.13 cm and that of female students was 24.70 ± 1.43 cm (p<0.001) (Table 1).

Pearson correlation coefficient was applied to evaluate the relationship of height with ulna length for the whole study population, for males and for females, and it was statistically significant for all three correlations (p<0.001). The regression coefficient and constant were 3.698 and 70.369 for males (p<0.001). The regression coefficient and constant were 5.617 and 18.562 for females (p<0.001). The regression coefficient and constant were 4.671 and 42.830 for the whole study population (p<0.001) (Table 2).

Regression equations which were derived from data analysis to estimate height from ulna length are as follows:

For whole study population: 42.830 + 4.671 (length of ulna).

For male study population: 70.369 + 3.698 (length of ulna).

For female study population: 18.562 + 5.617 (length of ulna).

**Table 1:** Age, height, and length of ulna

Variable	Males (n=50)	Females (n=50)	p-value
Age, years*	23.26 ± 1.45	23.48 ± 1.47	0.454
Mean height, cm*	171.70 ± 8.05	157.30 ± 8.22	<0.001
Height range, cm	159 – 187	145 – 169	-
Mean ulna length, cm*	27.40 ± 2.13	24.70 ± 1.43	<0.001
Ulna length range, cm	24 – 31	23 – 27	-

**Table 2:** Correlation coefficient, regression coefficient, and constant

Variable	Males (n=50)	Females (n=50)	Combined (n=100)
Correlation coefficient	0.977	0.979	0.971
Regression coefficient	3.698	5.617	4.671
Constant	70.369	18.562	42.830

## Discussion

In a specific population, adult males are considerably taller than adult females [9]. Similar results were observed in the current study. In the current study, mean ulna length was also considerably more in males than in females and similar results have been observed in the past [10-11]. The length of the ulna is significantly superior to arm span [12] and length of hand [13] for the prediction of height. In a previous study, researchers observed clear racial variation in the correlation of length of ulna and body height in Asian, White and Black populations [14].

As the relationship between the growth of limbs and thorax is affected by race, age, and gender of the individual, it is nearly impossible to devise a universal formula to estimate the height. Therefore, the need for formulating the population-specific as well as gender-specific formulae to estimate height has been highlighted by scientists. Regression models to estimate height from percutaneous ulna length [15], percutaneous length of the tibia [16], hand breadth [17], and hand length [17] have been devised after conducting studies in the past.

A study was conducted on the Iranian population and a significant relationship was observed of the dimensions of the upper limb with stature (p <0.05) [18]. Another recent study conducted on the Sudanese population [19] showed the results similar to those observed in our study and regression formulae for stature estimation were devised. It was observed in another study conducted on Bangladeshi adult females that age-related loss in stature can be assessed from the arm length and this relation is an alternative for the measurement of body stature [20]. Krishan and Sharma proposed linear as well as multiple regression to estimate body stature using measurements of hands and feet in the North Indian Rajput population [21]. Researchers in Maharashtra, India used the length of the hand and proposed a linear regression equation for the estimation of the stature in both genders [22]. They observed a significant relationship of the length of the hand with the body stature in both the genders and correlation coefficients were high, implying that this parameter can be used for estimation of height.

In our study, we are dealing with the interpretations of the correlation of an individual’s height and the average of the lengths of the right and left ulna. Ulna was chosen for this study because the length of the ulna is easier to measure more accurately in comparison with other bones of the upper limbs as it has clear bony markings. Formulae devised in our study to estimate height from ulna length showed similar precisions as was observed in a previous study. Mondal MK et al. [23] conducted a study on West Bengali males and observed the correlation coefficient to be 0.78 for right ulna and 0.68 for the left ulna. In the current study, we used the average of the lengths of the right and left ulna and observed the correlation coefficient to be 0.977 for males and 0.979 for the females. Our study showed results similar to those observed in the study conducted by Ilayperuma et al. [5].

## Conclusions

Length of ulna provides a reliable and relatively accurate means for estimating an individual’s height. Regression formulae devised will be of practical use to forensic scientists, anthropologists, archeologists, clinicians, and anatomists for estimating the stature from the length of the ulna.
